# Probing Genomic Aspects of the Multi-Host Pathogen *Clostridium perfringens* Reveals Significant Pangenome Diversity, and a Diverse Array of Virulence Factors

**DOI:** 10.3389/fmicb.2017.02485

**Published:** 2017-12-12

**Authors:** Raymond Kiu, Shabhonam Caim, Sarah Alexander, Purnima Pachori, Lindsay J. Hall

**Affiliations:** ^1^Gut Health and Food Safety, Quadram Institute Bioscience, Norwich Research Park, Norwich, United Kingdom; ^2^Norwich Medical School, University of East Anglia, Norwich Research Park, Norwich, United Kingdom; ^3^Public Health England, London, United Kingdom; ^4^Earlham Institute, Norwich Research Park, Norwich, United Kingdom

**Keywords:** *Clostridium perfringens*, pangenome, antimicrobial resistance, genomics, whole genome sequencing, Clostridial infection, exotoxins

## Abstract

*Clostridium perfringens* is an important cause of animal and human infections, however information about the genetic makeup of this pathogenic bacterium is currently limited. In this study, we sought to understand and characterise the genomic variation, pangenomic diversity, and key virulence traits of 56 *C. perfringens* strains which included 51 public, and 5 newly sequenced and annotated genomes using Whole Genome Sequencing. Our investigation revealed that *C. perfringens* has an “open” pangenome comprising 11667 genes and 12.6% of core genes, identified as the most divergent single-species Gram-positive bacterial pangenome currently reported. Our computational analyses also defined *C. perfringens* phylogeny (16S rRNA gene) in relation to some 25 *Clostridium* species, with *C. baratii* and *C. sardiniense* determined to be the closest relatives. Profiling virulence-associated factors confirmed presence of well-characterised *C. perfringens*-associated exotoxins genes including α-toxin (*plc*), enterotoxin (*cpe*), and Perfringolysin O (*pfo* or *pfoA*), although interestingly there did not appear to be a close correlation with encoded toxin type and disease phenotype. Furthermore, genomic analysis indicated significant horizontal gene transfer events as defined by presence of prophage genomes, and notably absence of CRISPR defence systems in >70% (40/56) of the strains. In relation to antimicrobial resistance mechanisms, tetracycline resistance genes (*tet*) and anti-defensins genes (*mprF*) were consistently detected *in silico* (*tet*: 75%; *mprF*: 100%). However, pre-antibiotic era strain genomes did not encode for *tet*, thus implying antimicrobial selective pressures in *C. perfringens* evolutionary history over the past 80 years. This study provides new genomic understanding of this genetically divergent multi-host bacterium, and further expands our knowledge on this medically and veterinary important pathogen.

## Introduction

*Clostridium perfringens* is a Gram-positive spore-forming anaerobe, best known as the causative agent for the tissue necrotic disease gas gangrene (also known as Clostridial myonecrosis). Notably, *C. perfringens* has also been widely associated with various intestinal diseases across human and animal species including: broiler necrotic enteritis (Keyburn et al., 2008), human food poisoning (Scallan et al., 2011), and preterm necrotising enterocolitis (Sim et al., 2015; Heida et al., 2016). Remarkably, it is reported to secrete >20 degradative toxins which constitute its primary arsenal to initiate histotoxic pathogenesis in both humans and animals (Uzal et al., 2014; Revitt-Mills et al., 2015). Intestinal-associated disease aetiology is characterised by rapid anaerobic proliferation in host tissue accompanied by *in vivo* production of several key pore-forming toxins including α-toxin, β-toxin, and β2-toxin, that consequently disrupt epithelial barrier function and induce histotoxic infections or tissue necrosis (Li and Mcclane, 2006). Common disease symptoms include pronounced diarrhoea (human adult food poisoning) (Mcclung, 1945), gaseous tissue necrosis (neonatal humans and animals) (Kosloske et al., 1978), and a haemorrhagic gut (neonatal horses) (Mehdizadeh Gohari et al., 2015).

*C. perfringens* was first isolated in the 1890s and named *Clostridium welchii* after its discoverer William Henry Welch (Welch and Nuttall, 1891). This gut pathogen is serotyped (or, specifically toxinotyped) clinically from A to E based upon the combination of “major toxins” (namely, α-toxin, β-toxin, ι-toxin, and ε-toxin) it encodes (Hassan et al., 2015). Multiplex PCR on the key toxin genes and Matrix Assisted Laser Desorption/Ionization time-of-flight mass (MALDI-TOF) spectrometry are the most common typing/identification methods in medical laboratories alongside conventional culture-based biochemical identification (Van Asten et al., 2009; Nagy et al., 2012).

Although this widespread gut pathogen has been studied and experimentally characterised over the past century, these studies have primarily focused on *C. perfringens*-associated toxins (Nagler, 1939; Ohno-Iwashita et al., 1986; Gibert et al., 1997; Stevens and Bryant, 2002). Notably, information on the entire pangenome [or, “the entire collection of gene families that are found in a given species” (Mcinerney et al., 2017)] of this pathogen is limited; to the date of analysis (April 2017) only 51 strains have been sequenced and made available publicly (Geer et al., 2010).

Previous comparative genomic studies on *C. perfringens* (up to 12 strains) indicated substantial genome variation within the pangenome (Hassan et al., 2015). Hence, we sought to construct the latest and largest pangenome of *C. perfringens* to probe evolutionary relationships and understand the genomic makeup of this bacterium. The *C. perfringens* genomes publicly available represent strains isolated from various diseased hosts including gas gangrene (human), food poisoning (human), necrotic enteritis (poultry), enterotoxaemia (sheep), and haemorrhagic enteritis (dogs and foals) from the past 100 years. This collection also represents the full range of *C. perfringens* toxinotypes (A–E). In this study, we also sequenced an additional 5 *C. perfringens* strains (as part of the NCTC3000 project^1^), for their historical significance (mostly isolated before 1960s), and thus included 56 genomes (including 51 genomes in NCBI databases) in our *in silico* investigation. Here we characterise genomic aspects of this medically and veterinary important pathogen, and through bioinformatic analysis of Whole Genome Sequencing (WGS) data identify virulence traits, define evolutionary relationships and functional annotations of the pangenome. Our analysis indicates that *C. perfringens* has a surprisingly diverse pangenome (core genes ~12.6%), potentially driven by horizontal gene transfer (HGT).

## Results

### Probing evolutionary relationships

**Figure 1:**
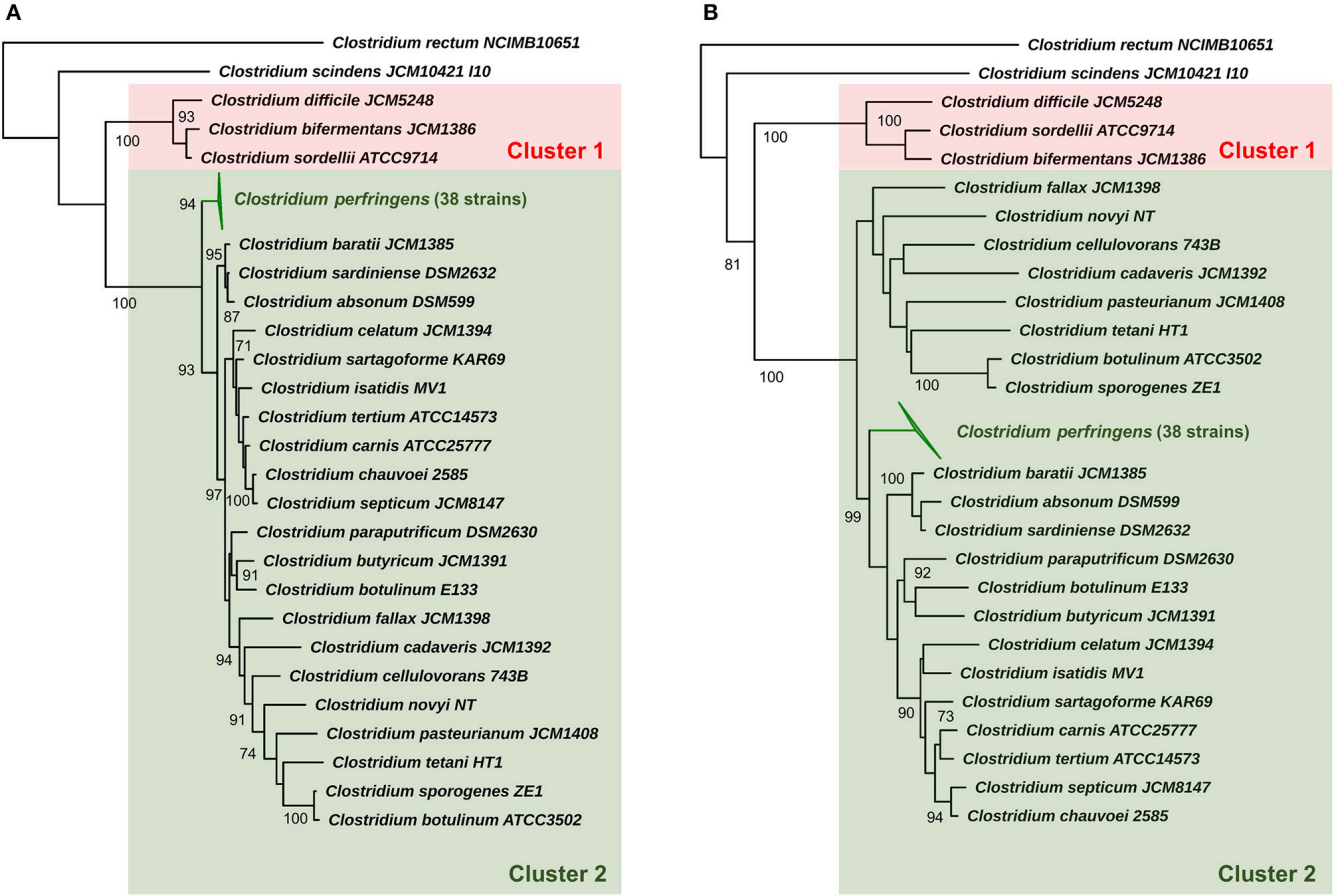
16S rRNA phylogeny of representative *Clostridium* species (25) and *C. perfringens*. **(A)** Maximum-likelihood phylogenetic tree of *C. perfringens* strains and representative *Clostridium* species (25) based on 16S rRNA genes with aLRT branch support values displayed. **(B)** Neighbour-joining phylogeny based on 16S rRNA genes supported by 1000 bootstrap replicates based on 1585 sites. Clusters 1 and 2 were assigned for description purposes. Branch support values >70 are shown on the nodes.

Initially we sought to determine taxonomy and phylogenetic relatedness between *C. perfringens* and a subset of pathogenic/environmental *Clostridium* species (25) using 16S rRNA approach (Supplementary Table 1) (Woese et al., 1990) as previous earlier studies had reported that *Clostridium pasteurianum, Clostridium baratii*, and *Clostridium absonum* were the closest relatives (Canard et al., 1992; Collins et al., 1994). Using the 16S rRNA gene predictor Barrnap (>800 bp sequences used, 38/56 strains) we determined that the 16S rRNA regions appear to be highly conserved (identity >99.1%; Supplementary Table 2) across all selected 38 *C. perfringens* strains as they form a monophyletic lineage in the 16S *Clostridium* phylogenetic tree. *C. perfringens* cluster was clearly separated from other *Clostridium* species, with closest relatives identified as *C. baratii* (toxin producer associated with infant botulism) and *Clostridium sardiniense* (α-toxin producers isolated from gas gangrene cases) (Figure 1) (Masaki et al., 1988; Harvey et al., 2002). Clinically important toxin-generating bacteria *C. botulinum*, potentially pathogenic *Clostridium paraputrificum, Clostridium tertium*, scavenger *Clostridium cadaveris*, and deadly-toxin producer *Clostridium tetani* all fall in the same sub-lineage (cluster 2) as *C. perfringens*, with *Clostridium difficile* (known for nosocomial antibiotic-associated diarrhea), *Clostridium sordellii* and *C. bifermentans* (uncommon environmental species infrequently linked to human diseases) diverging earlier from their common ancestor (cluster 1), suggesting an ancient divergence in evolutionary history (Edagiz et al., 2015).

Evolutionary distances based on near full-length 16S rRNA gene sequence (Table 1) also indicates that *C. baratii* and *C. sardiniense* are the closest relatives of *C. perfringens* in this dataset with sequence identities of ~95%, followed by *C. absonum* (94%), *C. paraputrificum* (93%), *C. septicum* (93%), *C. butyricum* (93%), *C. tetani* (91%), and *C. pasteurianum* (90%). These data support the 16S rRNA ML tree (Figure 1) as strains with higher sequence identities all appear to be more closely related to *C. perfringens* as indicated by tree topology.

**Table 1:** 16S rRNA sequence pair-wise comparison between five representative *C. perfringens* strains and 20 selected *Clostridium* species.

**Strains**	***Clostridium perfringens***									
	**ATCC13124 (1,511 bp)**		**Str13 (1,513 bp)**		**FORC003 (1,511 bp)**		**JP838 (1,511 bp)**		**NCTC2544 (1,511 bp)**	
	**Identity (%)**	**Coverage (%)**	**Identity (%)**	**Coverage (%)**	**Identity (%)**	**Coverage (%)**	**Identity (%)**	**Coverage (%)**	**Identity (%)**	**Coverage (%)**
*Clostridium baratii* JCM1385	95.33	100	95.06	100	95.33	100	95.26	100	95.33	100
*Clostridium sardiniense* DSM2632	95.18	99.67	94.92	99.80	95.05	99.67	95.11	99.67	95.18	99.67
*Clostridium absonum* DSM599	94.35	99.79	94.08	99.93	94.21	99.79	94.16	100	94.35	99.79
*Clostridium paraputrificum* DSM2630	93.77	99.93	93.52	100	93.77	99.93	93.91	100	93.64	99.93
*Clostridium sartagoforme* KAR69	93.73	99.86	93.60	100	93.66	99.86	93.73	99.86	93.59	99.86
*Clostridium septicum* JCM8147	93.70	100	93.57	100	93.77	100	93.63	100	93.56	100
*Clostridium isatidis* MV1	93.58	99.93	93.32	100	93.71	99.93	93.91	99.93	93.44	99.93
*Clostridium fallax* JCM1398	93.57	100	93.45	100	93.50	100	93.52	100	93.44	100
*Clostridium tertium* ATCC14573	93.53	100	93.25	100	93.46	100	93.53	100	93.38	100
*Clostridium botulinum* E133	93.45	100	91.09	100	93.52	100	93.67	100	93.59	100
*Clostridium butyricum* JCM1391	93.42	100	93.30	100	93.49	100	93.63	100	93.29	100
*Clostridium botulinum* Eklund17B	93.32	100	93.10	100	93.32	100	93.48	100	93.47	100
*Clostridium celatum* JCM1394	92.82	100	92.56	100	92.89	100	92.82	100	92.69	100
*Clostridium carnis* ATCC25777	92.57	99.86	92.30	100	92.50	99.86	92.57	99.86	92.43	99.86
*Clostridium cellulovorans* 743B	91.64	100	91.39	100	91.71	100	91.91	100	91.51	100
*Clostridium tetani* HT1	91.21	99.93	91.09	100	91.14	99.93	91.35	100	91.35	99.93
*Clostridium cadaveris* JCM1392	90.58	100	90.32	100	90.58	100	90.79	100	90.51	100
*Clostridium pasteurianum* JCM1408	90.22	100	90.10	100	90.29	100	90.28	100	90.09	100
*Clostridium difficile* JCM5248	86.25	100	86.14	100	86.32	100	86.39	100	86.25	100
*Clostridium bifermentans* JCM1386	85.82	100	85.68	100	85.89	100	85.89	100	85.82	100
*Clostridium rectum* NCIMB10651	79.66	100	79.60	100	79.66	100	79.63	100	79.57	100

### Genome description

A total of 51 genomes were retrieved in preassembled nucleotide FASTA files from NCBI database for further analysis (March 2017) and 5 NCTC strains were subjected to optimised DNA extraction procedures and sequenced (PacBio) as part of NCTC 3000 project (www.sanger.ac.uk/resources/downloads/bacteria/nctc/) with genomes annotated using Prokka. Whilst, contig number was variable (1–274) for NCBI-associated genomes (sequenced by different platforms and assembled using different methods), NCTC13170 and NCTC2544 reads assembled into 1 unitig, and NCTC8503, NCTC8797, and NCTC8678 genomes <10 contigs. Genome size of *C. perfringens* isolates ranged from 2.9 to 4.1 million bases, with an average GC content between 27.7 and 28.7% (Table 2). Predicted genes range from 2,600 to 3,800 with a median of 3304 genes.

**Table 2:** Genome description of 56 *C. perfringens* isolates included in this study.

	**Strain**	**Genome size (bp)**	**Contigs**	**Predicted genes**	**G+C (%)**
1	1207	3207161	95	2838	28.11
2	13	3085740	2	2827	28.51
3	2789STDY5608889	3321076	48	3000	28.08
4	ATCC13124	3256683	1	2973	28.38
5	B_ATCC3626	3896305	98	3563	28.36
6	C_JGS1495	3661329	84	3354	28.58
7	CP4	3642209	98	3469	27.80
8	CPE_F4969	3510272	74	3172	28.57
9	D_JGS1721	4045016	221	3768	28.26
10	E_JGS1987	4127102	101	3896	28.08
11	F262	3464306	53	3213	28.00
12	FORC003	3395109	2	3091	28.41
13	FORC025	3343822	1	3082	28.49
14	JFP718	3652220	56	3387	27.99
15	JFP727	3624033	47	3355	28.01
16	JFP728	3579626	85	3313	27.98
17	JFP771	3495308	81	3245	27.99
18	JFP774	3548595	56	3252	28.03
19	JFP795	3578912	67	3286	28.01
20	JFP796	3601148	114	3330	28.01
21	JFP801	3580610	59	3298	28.01
22	JFP804	3625968	117	3331	28.02
23	JFP810	3830406	95	3651	28.04
24	JFP826	3660408	70	3398	27.99
25	JFP828	3564370	54	3303	27.99
26	JFP829	3639686	108	3354	27.96
27	JFP833	3521770	96	3277	27.94
28	JFP834	3549140	121	3305	27.88
29	JFP836	3599532	51	3341	28.03
30	JFP914	3819232	79	3607	27.96
31	JFP916	3660641	67	3400	28.02
32	JFP921	3601767	78	3310	27.95
33	JFP922	3612099	76	3334	27.98
34	JFP923	3589715	48	3291	28.02
35	JFP941	3594640	64	3330	28.00
36	JFP961	3617219	66	3330	28.01
37	JFP978	3585277	68	3383	27.90
38	JFP980	3654588	67	3385	28.00
39	JFP981	3580367	68	3388	27.90
40	JFP982	3670557	62	3406	28.01
41	JFP983	3657522	46	3388	28.00
42	JFP986	3669049	58	3406	27.99
43	JFP992	3657868	70	3376	28.01
44	JJC	3259329	69	2970	28.12
45	JP55	3347300	1	3088	28.38
46	JP838	3530414	1	3244	28.38
47	MJR7757A	3585666	274	3265	27.79
48	NA	3417203	31	3157	28.33
49	NCTC8239	3324319	55	2942	28.66
50	SM101	2921996	3	2696	28.23
51	WAL14572	3462156	35	3264	28.10
52	NCTC13170	3310238	1	2993	28.36
53	NCTC2544	3195093	1	2854	28.46
54	NCTC8503	3577234	9	3294	28.20
55	NCTC8678	3005443	6	2785	28.12
56	NCTC8797	3021559	5	2813	28.21
Summary: Median(range)		3.58(2.92-4.12) Mb	65(1-274)	3304(2696-3896)	28.02(27.79-28.66)

### Pangenome construction and analysis

**Figure 2:**
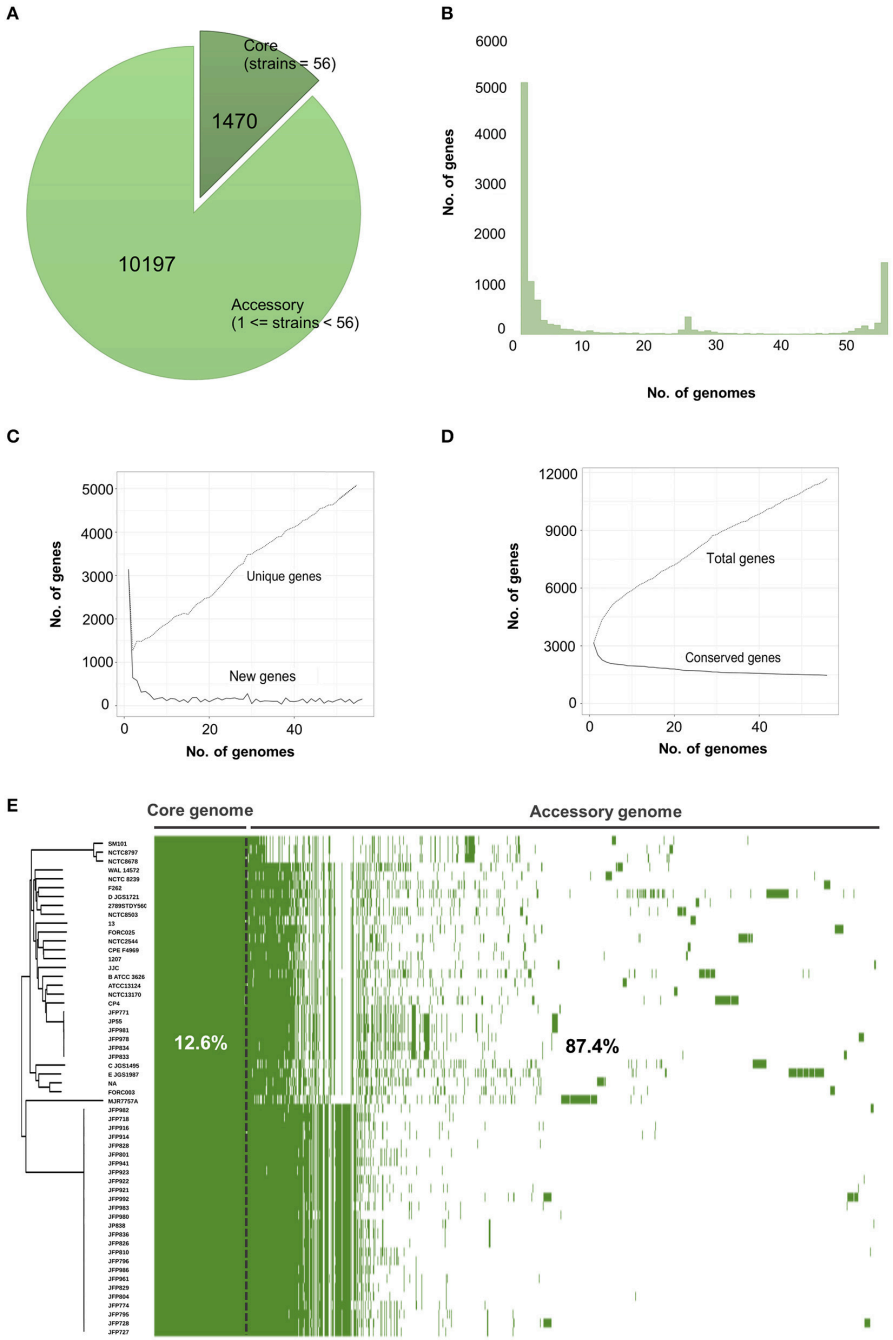
Visualisation and statistics of *C. perfringens* pangenome. **(A)** Core and accessory genes statistics. **(B)** Frequency bar graphs of number of identical genes against number of genomes. **(C)** Number of new genes and unique genes along pangenome computation. **(D)** Number of conserved genes and total genes along pangenome calculation. **(E)** Linearised pangenomic view of 56 *C. perfringens* isolates. Green cells represent syntenies aligned next to Neighbour-Joining core-genome based phylogenetic tree.

To define key genomic components, we next constructed the pangenome of *C. perfringens* encompassing 56 isolates, which represents the largest analysis of this type to date. This pangenome comprises 11,667 genes; 1470 core genes and 10,197 accessory genes (Figure 2A). Remarkably, only 12.6% represented core genes and a surprisingly high 44% (5139/11667) of unique genes (defined as genes only present in 1 strain in this pangenome; Figures 2B–E). This analysis implies very high genome plasticity in this pathogen, more than any other known prokaryotes currently published and studied (Mcinerney et al., 2017).

### Phylogeny and genome comparison

**Figure 3:**
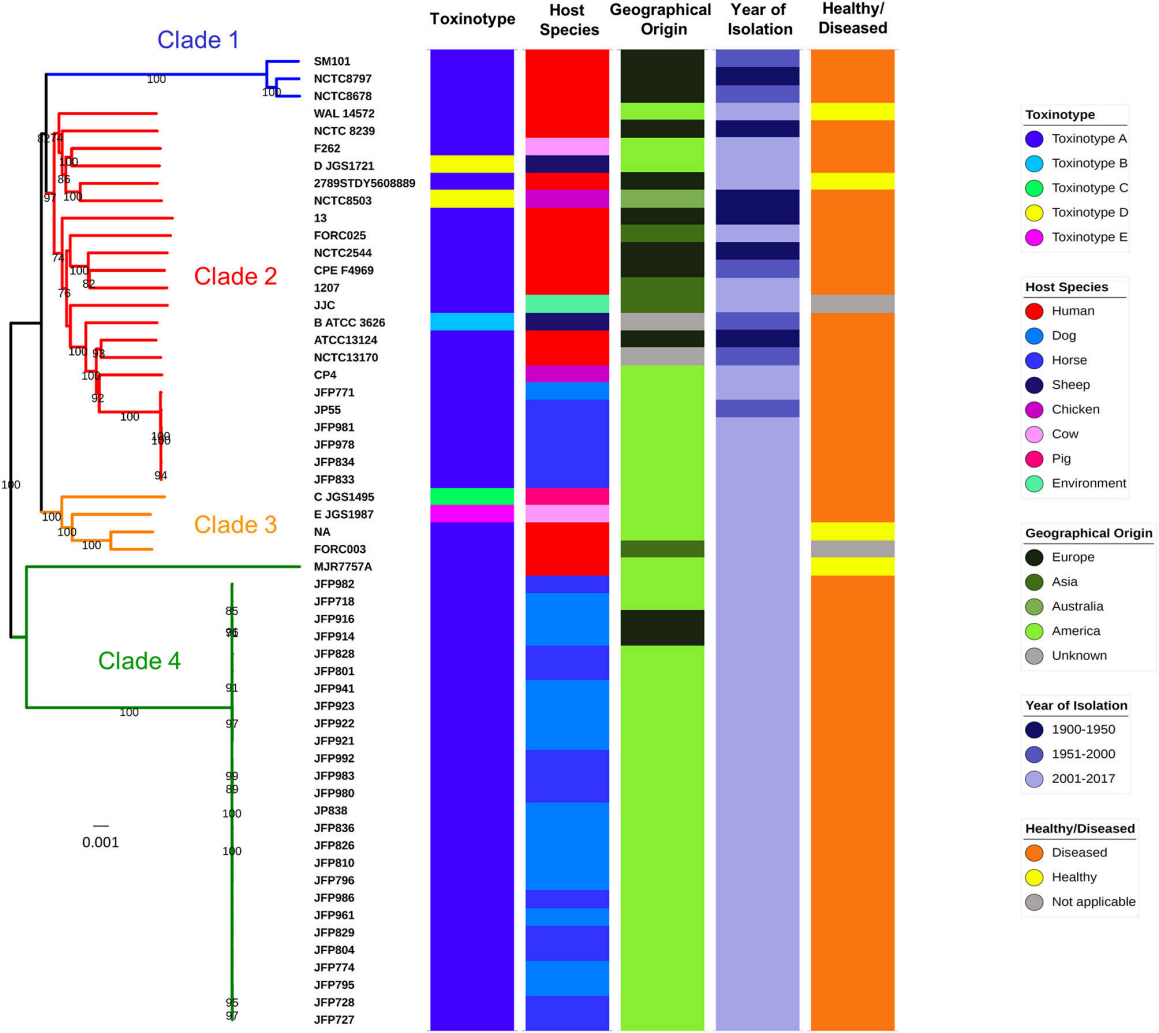
Genome comparison and core-genome phylogeny of *C. perfringens*. Core-genome based Neighbour-Joining (NJ) phylogeny of 56 isolates. Branch support values >70 (based on 1,000 bootstrap replicates) are shown on the nodes. Categories of metadata were displayed for comparison. MJR7757A is assigned to Clade 4 although it is distant from the rest of JFP isolates.

A core-genome phylogenetic approach was used for inference of *C. perfringens* genetic relatedness of these 56 isolates (Figure 3). The generated phylogenetic tree indicated four main clades, which correlated with pertinent metadata (Table 3). Food poisoning isolates SM101, NCTC8797, and NCTC8678 (collected between1940–60 from diseased human adults) clustered in a single lineage (clade 1), whereas human, sheep, chicken, dog, horse, and soil isolates intermingled in clade 2 or clade 3, suggesting potential spread and transmission between hosts. In clade 4 (clearly split from clade 1–3), most isolates were collected from a North American dog and horse *C. perfringens* haemorrhagic enteritis study (JFP isolates) indicating these isolates might be host- and disease-specific. This supports previous work where these JFP *netF*-positive isolates were found to cluster closely in a cgMLST tree (Mehdizadeh Gohari et al., 2017). Furthermore, the presence of 2 JFP isolates of Swiss origin also clustered in clade 4, which again suggests disease- and host- specificity, but exclusion of geographical linkages. We also observed that different toxinotypes grouped within the same clade (Figure 3 and Table 3). These findings are similar to those previously reported by Hassan et al. and Mehdizadeh Gohari et al. and suggest that certain toxin genes are encoded within the accessory genome (Li et al., 2013; Hassan et al., 2015; Park et al., 2016; Mehdizadeh Gohari et al., 2017). Our analysis also demonstrates the presence of several major toxins encoded within *C. perfringens* plasmids (sequences individually retrieved from NCBI nucleotide database), highlighting the potential for toxin gene transfer via HGT (Supplementary Figure 1). Interestingly, whole-genome alignment-free phylogeny (Supplementary Figure 2) shows the clustering of toxinotypes (B-E in 1 lineage), in contrast to the core-genome based phylogeny (Figure 3), which further supports the hypothesis that several specific major toxins obtained via HGT are present in accessory genome.

**Table 3:** Metadata of the *C. perfringens* genomes used for analysis in this study.

	**Isolate**	**Alternative name**	**Toxino-type**	Year of isolation^*^	**Origin of isolates**	**Host diseased states**	**NCBI accession**	**References**
1	ATCC13124	NCTC8237	A	1941	Human	Gas gangrene	SAMN02604008	Möllby et al., 1976
2	SM101	NCTC8798	A	1953	Meat rissole/Human	Food poisoning	SAMN02604026	Hobbs et al., 1953; Myers et al., 2006
3	Strain 13	Lechien	A	1939	Human	Gas gangrene	SAMD00061119	Paquette and Fredette, 1967; Mahony and Moore, 1976; Shimizu et al., 2002
4	FORC_003	FORC3	A	2015	Contaminated food	n/a	SAMN03140316	n/a
5	JP55	JFP55	A	1999	Foal	Necrotizing enteritis	SAMN03372134	Mehdizadeh Gohari et al., 2016
6	JP838	JFP838	A	2009	Dog	Haemorrhagic gastroenteritis	SAMN03377063	Mehdizadeh Gohari et al., 2016
7	FORC_025	FORC25	A	2016	Human	Food poisoning	SAMN04209542	n/a
8	F262		A	2011	Calf	Bovine Clostridial abomasitis	SAMN00199225	Nowell et al., 2012
9	WAL-14572		A	2012	Human (gut of an autistic child)	Human Microbiome Project	SAMN02463856	Ribeiro et al., 2012
10	CP4		A	2006	Chicken	Necrotic enteritis	SAMN04017578	Lepp et al., 2010
11	MJR7757A		A	2016	Human (vagina)	Human Microbiome Project	SAMN03842618	Ribeiro et al., 2012
12	JGS1495		C	2007^*^	Pig	Diarrhoea	SAMN02436294	Lepp et al., 2010
13	ATCC3626	NCTC13110, NCIMB 10691	B	1955	Lamb	Dysentery	SAMN02436295	Lepp et al., 2010
14	JGS1987		E	2007^*^	Calf	Haemorrhagic Enteritis	SAMN02436167	Lepp et al., 2010
15	F4969		A	1995	Human	Diarrhoea	SAMN02436168	Cornillot et al., 1995
16	NCTC8239	ATCC12917	A	1949	Salt beef/ Human	Food poisoning	SAMN02436239	Hassan et al., 2015
17	JGS1721		D	2008^*^	Sheep	Enterotoxaemia	SAMN02436277	Hassan et al., 2015
18	JJC		A	2013	Landfill sludge	n/a	SAMN02317206	Wong et al., 2014
19	2789STDY5608889		A	2015^*^	Human	Healthy donor	SAMEA3545297	n/a
20	JFP718		A	2011	Dog	Haemorrhagic gastroenteritis	SAMN05323879	Mehdizadeh Gohari et al., 2017
21	JFP774		A	2011	Dog	Haemorrhagic gastroenteritis	SAMN05323883	Mehdizadeh Gohari et al., 2017
22	JFP728		A	2011	Foal	Necrotizing enteritis	SAMN05323881	Mehdizadeh Gohari et al., 2017
23	JFP727		A	2011	Foal	Necrotizing enteritis	SAMN05323880	Mehdizadeh Gohari et al., 2017
24	JFP795		A	2012	Dog	Haemorrhagic gastroenteritis	SAMN05323884	Mehdizadeh Gohari et al., 2017
25	JFP801		A	2002	Foal	Necrotizing enteritis	SAMN05323886	Mehdizadeh Gohari et al., 2017
26	JFP804		A	2010	Foal	Necrotizing enteritis	SAMN05323887	Mehdizadeh Gohari et al., 2017
27	JFP796		A	2012	Dog	Haemorrhagic gastroenteritis	SAMN05323885	Mehdizadeh Gohari et al., 2017
28	JFP771		A	2011	Dog	Haemorrhagic gastroenteritis	SAMN05323882	Mehdizadeh Gohari et al., 2017
29	JFP810		A	2012	Dog	Haemorrhagic gastroenteritis	SAMN05323888	Mehdizadeh Gohari et al., 2017
30	JFP826		A	2012	Dog	Haemorrhagic gastroenteritis	SAMN05323889	Mehdizadeh Gohari et al., 2017
31	JFP828		A	2011	Foal	Necrotizing enteritis	SAMN05323890	Mehdizadeh Gohari et al., 2017
32	JFP829		A	2010	Foal	Necrotizing enteritis	SAMN05323891	Mehdizadeh Gohari et al., 2017
33	JFP833		A	2000	Foal	Necrotizing enteritis	SAMN05323892	Mehdizadeh Gohari et al., 2017
34	JFP834		A	2002	Foal	Necrotizing enteritis	SAMN05323893	Mehdizadeh Gohari et al., 2017
35	JFP836		A	2008	Dog	Haemorrhagic gastroenteritis	SAMN05323894	Mehdizadeh Gohari et al., 2017
36	JFP914		A	2009	Dog	Haemorrhagic gastroenteritis	SAMN05323895	Mehdizadeh Gohari et al., 2017
37	JFP916		A	2009	Dog	Haemorrhagic gastroenteritis	SAMN05323896	Mehdizadeh Gohari et al., 2017
38	JFP921		A	2007	Dog	Haemorrhagic gastroenteritis	SAMN05323897	Mehdizadeh Gohari et al., 2017
39	JFP922		A	2006	Dog	Haemorrhagic gastroenteritis	SAMN05323898	Mehdizadeh Gohari et al., 2017
40	JFP923		A	2006	Dog	Haemorrhagic gastroenteritis	SAMN05323899	Mehdizadeh Gohari et al., 2017
41	JFP941		A	2013	Dog	Haemorrhagic gastroenteritis	SAMN05323900	Mehdizadeh Gohari et al., 2017
42	JFP961		A	2013	Dog	Haemorrhagic gastroenteritis	SAMN05323901	Mehdizadeh Gohari et al., 2017
43	JFP978		A	2011	Foal	Necrotizing enteritis	SAMN05323902	Mehdizadeh Gohari et al., 2017
44	JFP980		A	2006	Foal	Necrotizing enteritis	SAMN05323903	Mehdizadeh Gohari et al., 2017
45	JFP981		A	2004	Foal	Necrotizing enteritis	SAMN05323904	Mehdizadeh Gohari et al., 2017
46	JFP982		A	2001	Foal	Necrotizing enteritis	SAMN05323905	Mehdizadeh Gohari et al., 2017
47	JFP983		A	2004	Foal	Necrotizing enteritis	SAMN05323906	Mehdizadeh Gohari et al., 2017
48	JFP992		A	2008	Foal	Necrotizing enteritis	SAMN05323908	Mehdizadeh Gohari et al., 2017
49	JFP986		A	2011	Foal	Necrotizing enteritis	SAMN05323907	Mehdizadeh Gohari et al., 2017
50	1207_CPER		A	2012	Human (blood)	ICU patient	SAMN03197169	Roach et al., 2015
51	NA (not available)		A	2013	Human/infant	Premature infant	SAMN05509317	Raveh-Sadka et al., 2015
52	NCTC8678	ATCC12919	A	1951	Human	Food poisoning	SAMEA3867459	Hobbs et al., 1953
53	NCTC8797	HF2985/50	A	1950	Salt beef	Food poisoning	SAMEA3867461	Hobbs et al., 1953
54	NCTC13170	DSMZ 100947/ D10	A	1993	Human	Food poisoning	SAMEA3867463	Mooijman et al., 2003
55	NCTC8503	CN366	D	1930	Chicken	Necrotic enteritis	SAMEA3879480	Bennetts, 1932
56	NCTC2544	STEELE COL 5	A	1928	Human	Infected gall bladder	SAMEA3919787	n/a

* *Year of isolation here is predicted based on sequence submission date on public genome databases due to non-traceability in literature*.

Multi-dimensional scaling (MDS) investigation (Supplementary Figure 3) based on whole-genome gene-absence-presence phylogeny (Supplementary Figure 4) also mirrors the core-genome phylogeny findings (Figure 3); food-poisoning isolates cluster tightly, JFP isolates nest in 2 distinct lineages (clades 2 and 4) and remaining isolates form another cluster. Using JP838 as our reference genome (as it contains a large 3.5 Mbp chromosome for nucleotide comparison) we generated a circular genome comparison figure, based on 1–3 strains from each of the 4 core-genome clades (Supplementary Figure 5). This comparison supports our results from the pangenomic analysis highlighting significant variation; faded areas (<80% sequence similarity) exist in several regions as well as regions (>10) with differences in GC content, which may indicate genomic island insertion sites via HGT. Interestingly, an additional whole-genome search on strain JP838 indicated that most coding sequences predicted in divergent GC regions encode for hypothetical protein genes (via BLAST NR database) of unknown function.

### Functional annotation of core and accessory genomes

**Figure 4:**
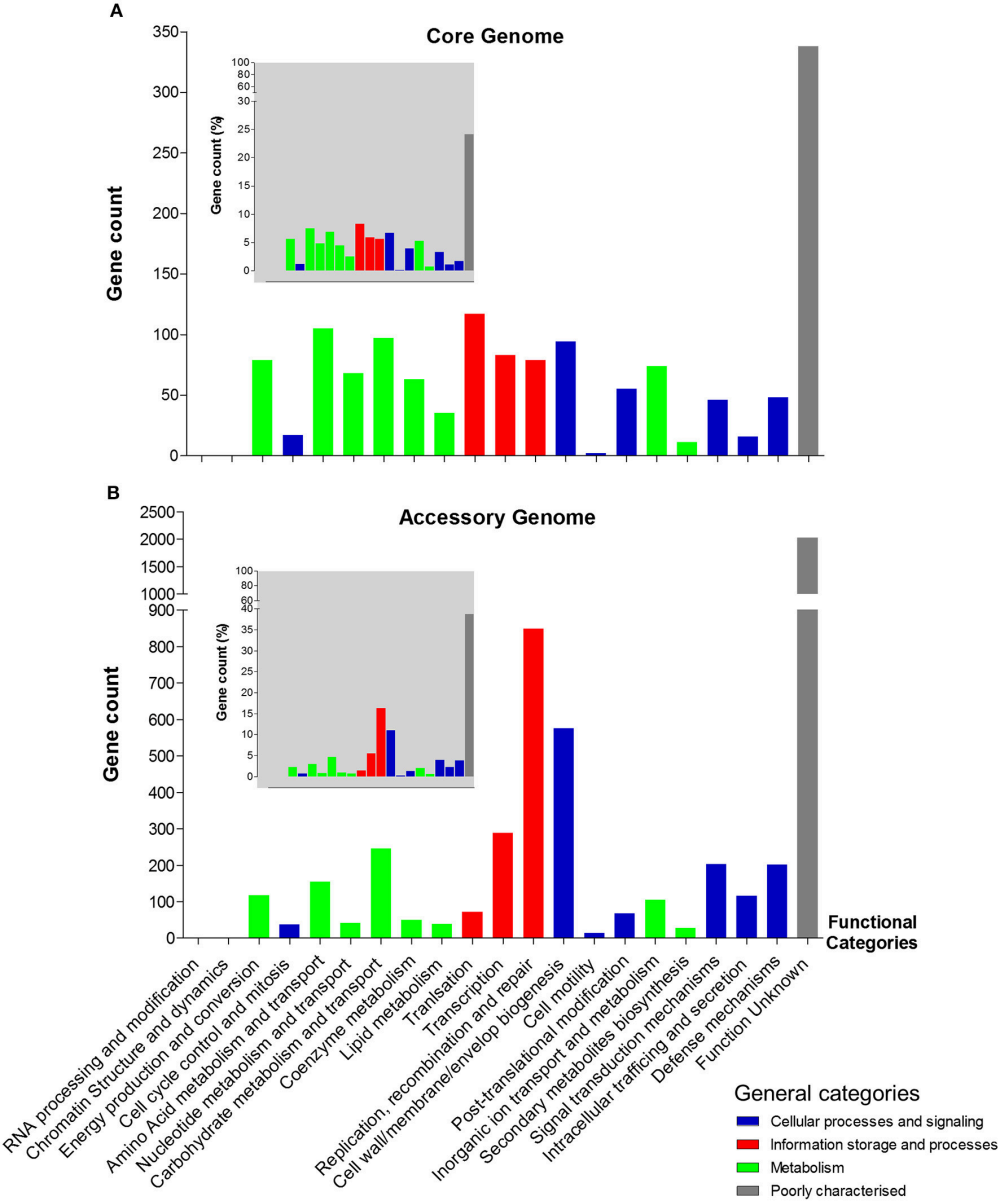
Functional annotation (COG) of *C. perfringens* core and accessory genomes. **(A)** Core genome functional annotation. **(B)** Accessory genome functional annotation. The smaller-scale grey-background charts show the percentage of gene count of each functional class in both core and accessory genomes corresponding to the functional categories. Non-matching genes (unclassified) are not included in the statistics.

We next analysed both the core and accessory genomes at a functional level using “Clusters of Orthologous Groups of proteins” (COG) and “Evolutionary Genealogy of Genes: Non-supervised Orthologous Groups” (eggNOG) databases (Galperin et al., 2015; Huerta-Cepas et al., 2016), to probe potential *C. perfringens* host adaption and/or pathogenesis traits (Figure 4).

Interestingly, 849 genes were assigned to protein families associated with “replication, recombination, and repair” comprising mainly transposases (*n* = 254), integrases (*n* = 31), and phage proteins (*n* = 7) within the accessory genome. Whereas, only 79 genes encoded within the core genome were assigned to this functional class, suggesting a key difference between both genomes at a functional level (849 vs. 79; 16.2 vs. 5.6%). Genes associated with “defense mechanisms” were higher within the accessory genome (*n* = 202), when compared to the core genome (*n* = 48), which includes genes encoding efflux pumps, restriction enzymes and ABC transporters (linked with iron uptake systems). Genes involved in metabolism (including carbohydrate, amino acid, and lipid) are proportionately more represented within the core genome (16.8%) vs. the accessory genome (8.3%), whilst recombination and cell-wall biogenesis related genes dominate the functionality of the accessory genome (27.1% vs. core genome: 12.2%). Notably, as indicated above there is a high proportion of unknown-function genes observed in both genomes (core: 24% vs. accessory: 38.7%; Figure 4).

### *In Silico* profiling of virulence traits

**Figure 5:**
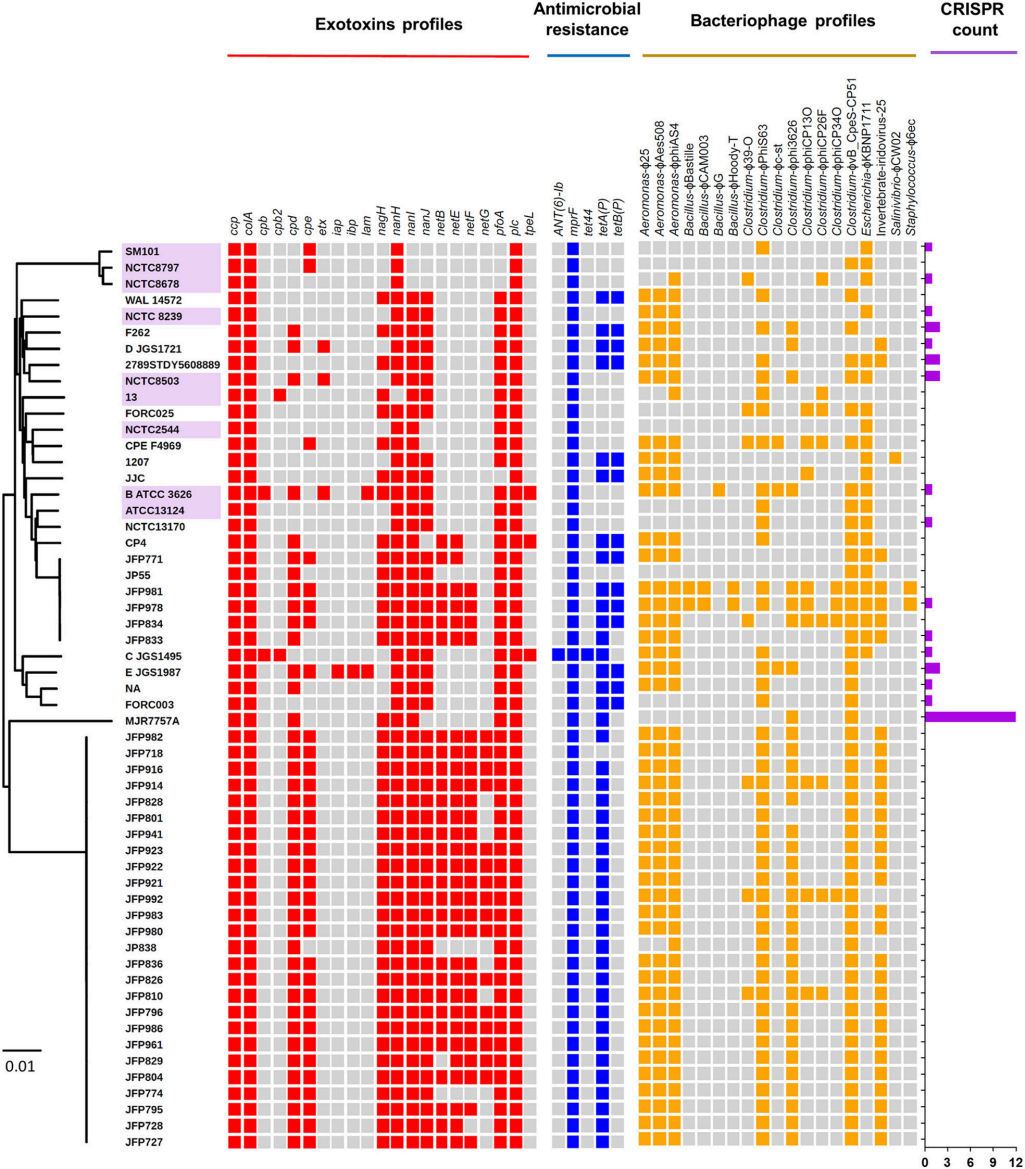
Virulence-associated potentials in *C. perfringens* genomes and plasmids. Heat-map visualising exotoxins, antimicrobial resistance, prophage profiles, and CRISPR counts of 56 *C. perfringens* genomes. Presence of genes are indicated by cell colours: grey (absence); red, blue, and yellow (presence). Purple-labelled isolates indicate historical isolates (pre-antibiotic era).

Toxins secreted by *C. perfringens* have long been considered key virulence factors and are implicated in multiple clinical disease phenotypes (Welch and Nuttall, 1891; Rood, 1998). Primary toxins produced include α-toxin, β-toxin, ι-toxin, and ε-toxins, which are used to classify this pathogen into different toxinotypes (Petit et al., 1999). These toxins, as well as additional toxins β2-toxin and perfringolysin O, potentially initiate pathogenesis through pore-forming and cytolysis (Supplementary Table 4). Therefore, we analysed the toxin profiles of the 56 isolates, and additional virulence-associated factors such as antimicrobial resistance (AMR) and prophage content (Figure 5).

Several toxins, including α-toxin (*plc*), α-clostripain (*ccp*), and microbial collagenase (*colA*) genes are conserved in all isolates (Figure 5). Other toxins, such as enterotoxin (*cpe*), a food-poisoning-linked toxin, was found to be encoded in food-poisoning isolates SM101 and NCTC8797, however this gene is also present in all JFP isolates (haemorrhagic enteritis isolates). Perfringolysin O (*pfoA* or *pfo*), a well-characterised pore-forming toxin, is encoded in most genomes except food-poisoning and JJC environmental isolates and presence of sialidase genes (*nan* and *nag*) vary across the isolates while *netB, netE, netF*, and *netG* genes were found to be uniquely encoded within JFP isolates (associated with haemorrhagic enteritis in dogs and foals), as reported previously (Mehdizadeh Gohari et al., 2016).

Antimicrobial-peptide (AMP) resistance gene multi-peptide resistance factor (*mprF*) was detected in all isolates. This gene is highly-conserved in certain Gram-positive and Gram-negative bacteria (Figure 5) and is known to confer resistance through modifying surface molecules of AMPs, including host defensins, and potentially also aminoglycoside antibiotics (Ernst and Peschel, 2011; Cole and Nizet, 2016). Tetracycline-resistant efflux protein *tetA(P)* gene (Bannam et al., 2004) were detected in 75% of the isolates (*n* = 42), and 23% of the isolates (*n* = 14) harboured double *tet*-resistance genes—*tetA(P)* and *tetB(P)*. The only other AMR-associated gene detected was specific aminoglycosides resistance gene (*ANT(6)-Ib*), which was encoded within 1 toxinotype C porcine isolate JGS1495.

Finally, genome scanning for prophage elements indicated that 19 in total were encoded, potentially derived from a variety of bacterial genera including *Aeromonas, Bacillus, Clostridium, Escherichia*, and *Staphylococcus* (Figure 5). Notably, JFP isolates also displayed unique prophage profiles including *Clostridium* phage ϕvB_cpes_CP51 (*n* = 48), *Aeromonas* phage ϕphiAS4 (*n* = 47), ϕaes508 and ϕ25 (*n* = 45), and *Clostridium* phage ϕphiS63 (*n* = 44). Interestingly, 40/56 (71.5%) isolates do not encode CRISPR system at all (*in silico* prediction = 0; Figure 5).

## Discussion

Although *C. perfringens* is an important causative agent of animal and human infections; our knowledge on the genome content and phylogenic characteristics of this pathogenic bacterium is currently limited. Our current study, analysing the largest number of *C. perfringens* genomes to date, highlights significant genomic plasticity, and an arsenal of key virulence factors, including toxins and AMR genes, that are encoded by this pathogen.

Using the 16S rRNA gene to initially probe *C. perfringens* phylogenic relationships (Koch et al., 1994), we observed clear deviation of 16S alignment from other *Clostridium* species (Figure 1), with *C. baratii* and *C. sardiniense* as the closest relatives of *C. perfringens* (16S sequence similarity ~95.3 and 95.1%), which agrees with previous 16S phylogenetic studies (Collins et al., 1994; Vos et al., 2009). We also performed alignment-free whole-genome-based phylogenetics, which has been reported to represent a more comprehensive and robust method for clustering genetically variable prokaryotes including *Helicobacter pylori* (Van Vliet and Kusters, 2015; Bernard et al., 2016). Our analysis indicates that this approach can also be used for *C. perfringens*, as we observed near-identical phylogeny when compared to the core-genome based method (Supplementary Figure 2), and may prove useful in epidemiology and outbreak management.

Previous limited genomic studies have indicated that *C. perfringens* is a genetically diverse organism, and our larger scale study probing 56 genomes also supports this notion. We determined that *C. perfringens* has a highly variable open/infinite pangenome, with new genes potentially being added at an average of 106 genes for each new genome sequenced (Supplementary Figure 6), which is in comparison to other pathogens, including *Yersinia pestis* (Eppinger et al., 2010) and *Bacillus anthracis* (Rouli et al., 2015), which have closed pangenomes. The “lifestyle” of the organism is proposed to reflect the nature of the pangenome, and *C. perfringens'* ability to thrive in multiple host species and environmental niches, including the human and animal gut and soil, allows active interaction with other bacterial species, and links to our open pangenome data (Rouli et al., 2015). To our knowledge, only a handful of bacterial pangenome studies have reported extreme pan-genomic variation. This trait (12.5% core genes) is an uncommon observation in other bacteria and is significantly lower than other reported prokaryotic species including *C. difficile* (30.3%) (Knight et al., 2016), *Streptococcus pneumoniae* (46.5%) (Hiller et al., 2007), *Salmonella enterica* (16%), and *Klebsiella pneumoniae* (26%) (Mcinerney et al., 2017). It should be noted that this pangenome analysis may be limited in terms of capturing total *C. perfringens* diversity due to the fact that 30 isolates in our analysis are from a single study (Mehdizadeh Gohari et al., 2017). These *netF*-positive isolates were sequenced and assembled for plasmid pathogenicity analysis, and were reported to have tight clonal relationship using a MLST approach that could potentially bias the analysis of this dataset. Thus, we expect that due to *C. perfringens'* wide host and environmental range, additional genetic variation potentially exists, and that a significantly larger-scale study, encompassing a greater variety and number of isolates from multiple ecological niches, would be required to obtain higher-resolution evolutionary insights.

*C. perfringens* is recognised to be the most prolific toxin-producing organism currently characterised, and is reported to produce >20 exotoxins. However, not all strains produce all toxins, and certain toxins are encoded on plasmids rather than chromosomes (Supplementary Figure 1). In our analysis, we showed that individual strains produce a diverse profile of toxins, with the exception of the JFP isolates, which display a unique pattern of toxin gene profiles (netE, netF, and netG), inferring that these encoded toxins may be associated with specific hosts (i.e., dogs and horses), and/or disease (i.e., haemorrhagic enteritis) (Mehdizadeh Gohari et al., 2016). Although poultry necrotic enteritis (NE) is thought to be mediated through the *netB* toxin, the NE isolate NCTC8503 was not found to encode this gene, which confirms a previous study by Brady et al. who determined that *netB* (via PCR testing) was present in both healthy and diseased chickens (Brady et al., 2010). Interestingly, *netB* was detected in 87.5% JFP isolates (28/32), suggesting that netB toxin might be involved in haemorrhagic enteritis in dogs and horses. Whist our analysis provides interesting linkages with toxin-associated genes with disease and host phenotypes, determination of specific associations will require further WGS data from a wider variety of isolates (including from healthy hosts), and confirmation from experimental studies.

As *C. perfringens* produces this vast array of toxins, a combination of several “key toxins” are traditionally used to type this species. However, our understanding behind the disease-toxinotype link is limited at present, in part due to lack of in-depth WGS studies. We observed that most of the disease-causing isolates in this dataset are toxinotype A (88%; 44/50), and are associated with diverse diseases in different hosts, suggesting the need to review current toxinotyping methods to reflect *C. perfringens* genetic and pathogenesis relationships more closely. From a public health standpoint, a reliable typing method is also necessary to track the spread of bacterial pathogens, and outbreaks. Currently, there is a move from traditional serotyping and MLST approaches to more recent WGS core-genome based methods, which are employed by national-level health department including Public Health England in the UK (Ashton et al., 2016). Notably, phylogenomic clustering (using CVTree) resembles the core-genome tree, indicating the possibility of employing phylogenomic approaches for large dataset epidemic analysis, as it uses significantly less computational resources (Bernard et al., 2016).

The extreme pan-genomic variation of *C. perfringens*, appears to be driven by HGT events, as indicated by the high number of genes associated with transposases, integrases, and phages, which infers the high potential for gene gain or loss events (Figure 4). Notably, *C. perfringens* is found in multiple ecological niches, which are associated with diverse microbial (including viral) communities. Genomic integration of prophages seemingly represents a key source of genetic diversity in *C. perfringens*, and prophage diversity in these isolates surpass the phage contents of *C. difficile*; 7 prophages were found in a total of 44 isolates (Knight et al., 2016). Notably, phages in the human gut are reported to outnumber bacteria by at least a factor of 10, and their lysogenic lifestyle has a powerful biological impact on its hosts such as modifying the fitness and metabolism of bacterial hosts (Brüssow and Hendrix, 2002). Bacteriophages were reported to boost the sporulation capacity of *C. perfringens* in several studies, which reflects the survival strategy employed by these viral particles (Stewart and Johnson, 1977). Some prophage-like genes were also associated with toxin secretion in *C. difficile* (Goh et al., 2005), *Salmonella enterica* (Thomson et al., 2004), and *E. coli* (Zhang et al., 2000). Thus, diverse prophage-associated gene integration in *C. perfringens* genomes may be central for our understanding of the pathogenicity of *C. perfringens* in terms of human and animal health. This high prophage genome plasticity is further supported by our CRISPR (Clustered regularly interspaced short palindromic repeats) array investigation on these 56 isolates as 71.5% (40/56) are predicted to possess no CRISPR system (count = 0), which encodes systems to defend against viral/phage invasion (Figure 5). Interestingly, genes involved in “defense mechanisms” are encoded at higher numbers within the accessory genome (*n* = 202), than the core genome (*n* = 48), again suggesting constant genomic adaptation to environmental change. These include genes encoding multidrug efflux pumps (associated with antibiotic resistance; *n* = 11) and ABC transporters (linked with iron-uptake mechanisms and multi-drug resistance; *n* = 83) (Köster, 2001; Andersen et al., 2015). Notably, genes encoding functions expected to be conserved, such as metabolism (i.e., carbohydrate, amino acid, and lipid metabolism) are more represented within the core (16.8%) rather than the accessory genome (8.3%), indicating their critical functionality in terms of colonisation and growth in different nutritional environments.

There is an ever-increasing incidence of multi-drug resistance pathogens that now pose a critical risk to human and animal health. AMR within *C. perfringens* strains is a serious concern due to its ability to encode multiple virulence associated factors e.g. toxins, that are linked to severe disease. Previous phenotyping-based studies have reported *C. perfringens* as a multidrug resistant pathogen (resist against >1 antimicrobial drug) (Tansuphasiri et al., 2005). However, our genomic analysis indicates that tetracycline resistance genes are the only AMR-specific genes that are wide-spread in this pathogen, which corresponds to previous findings of phenotypically observed tetracycline resistance in *C. perfringens* strains isolated from pigs, human, and environments (Rood et al., 1978). Resistance genes *tetA(P)* and *tetB(P)* are both tetracycline efflux proteins most commonly known to be encoded in *C. perfringens* since the 1980s (Lyras and Rood, 1996). Notably, our data indicates that strains isolated prior to the 1950s do not encode any *tet* genes, which may correlate with the fact that tetracycline did not come into commercial use until 1978, supporting the emergence of antimicrobial resistance under selective pressure in the post-antibiotic era. Although *C. perfringens* does encode tetracycline resistance-associated genes, this is a limited AMR profile when compared to other Gram-positive enteric pathogens like *C. difficile* (Knight et al., 2016). Novel/uncharacterised AMR genes and transmission route (directly from environments such as soil) may account for these findings, however a larger dataset from hospital and/or farming environments and phenotypic testing is required to confirm this hypothesis. Nevertheless, it is suggestive that efflux pumps families including efflux pumps (e.g., *mepA*) and ATP-Binding Cassette (ABC) transporters were *in silico* detected as these could potentially play a significant role in multidrug resistance as observed in previous clinical cases (Andersen et al., 2015). Furthermore, the environment in which *C. perfringens* resides (e.g., the gut) could also potentially play a significant role in the acquisition of new AMR genes via the “resistome,” and as such warrants further investigations (Browne et al., 2017). The host also has specific mechanisms for directly killing gut-associated bacteria including via AMPs. Interestingly, the *mprF* gene (known to be involved in resistance to human defensins) was found to be conserved in all *C. perfringens* genomes, which may link to its ability to evade host-associated defenses for gut colonisation. Indeed, previous studies in *Staphylococcus aureus* have indicated that *mprF* mutant strains are more prone to mammalian AMP killing including neutrophil defensins (Peschel et al., 2001), and mutants are less virulent in animal infection models (Kristian et al., 2003). Importantly, homologues of *mprF* are also reported to dampen the effectiveness of certain clinical antimicrobials (aminoglycosides) such as vancomycin and gentamicin (Nishi et al., 2004), and this may link to previous studies showing resistance of *C. perfringens* to these antibiotics (Traub and Raymond, 1971; Tyrrell et al., 2006; Osman and Elhariri, 2013).

## Conclusion

High-throughput WGS has paved the way for in-depth pangenomic studies into bacterial species, however previous studies on *C. perfringens* have utilised a maximum of 12 strains for analysis, and thus genomic information on this pathogen is currently limited. Our analysis of 56 strains indicates a surprisingly diverse pangenome, and *in silico* functional analysis reveals frequent exchange of genes in its evolutionary history, associated with prophages, toxins and AMR genes, that potentially influence pathogenicity. Interestingly, this study did not correlate any toxinotype to a specific disease in phylogenetic clustering, however a larger dataset may be needed to establish any causative relationships. As most of the isolates in our study are from diseased patients/animals, inclusion of isolates from healthy samples may enable us to understand the genomic signatures that separate commensal from pathogenic *C. perfringens* (Nagpal et al., 2015). Whilst these genomic studies are of central importance it is vital to also determine the phenotypic disease-causing mechanisms, thus experimental studies are essential to probe our understanding of *C. perfringens* pathogenesis in tandem with *in silico* exploration.

## Materials and methods

### Bacterial isolates and metadata

*C. perfringens* isolates previously sequenced and deposited in NCBI were used in this study (*n* = 51; retrieved in March 2017), a further 5 *C. perfringens* isolates (NCTC8678, NCTC8797, NCTC13170, NCTC2544, and NCTC8503) were sequenced as part of the NCTC 3000 sequencing project to be included in this study. Metadata of all 56 isolates were retrieved manually online (March 2017) either from published literature, National Center of Biological Information (NCBI) databases or European Bioinformatics Institute (EBI) databases. Year of isolation and geographical origins of isolates were taken from published research papers, otherwise estimated using dates/locations on public databases as indicated. WGS accessions, references and metadata for all genomes are provided in the Table 3.

### Genomic DNA extraction and whole genome sequencing

For genomic DNA extraction, *C. perfringens* strains (NCTC8678, NCTC8797, NCTC13170, NCTC2544, and NCTC8503) were propagated on blood agar at 37°C for 36 h under anaerobic conditions. Bacterial lysates were prepared in buffer B1 (containing additional RNase A, protease, and Lysozyme) and the lysates were incubated at 80°C for 24 h. Post-incubation high Molecular weight DNA was isolated from *C. perfringens* strains using the Qiagen midi kit. DNA quality (>60 kb) and quantity (>3 μg) was assessed using the Agilent 2200 TapeStation and Qubit® dsDNA BR Assay Kit respectively. WGS was performed using the PacBio SMRT® DNA Sequencing technology. Appropriate biosafety procedures were in place for both bacterial culturing and DNA extraction.

### *De Novo* assembly and annotation

Assembled genomes (51) previously sequenced were retrieved online on NCBI Genomes database (Geer et al., 2010). All 56 genomes were annotated using Prokka v1.10 (Seemann, 2014). HGAP.3 from SMRTpipe-2.3.0 was used to perform *de novo* assembly of the 5 strains of *C. perfringens* sequenced on PacBio RS I obtained from ENA, with one SMRT cell per strain assembled into unitigs. The minlongreadlength (assembly seed length) parameter was set such that there was 30X genome coverage above the seed length. Two strains (NCTC13170 and NCTC2544) were assembled into one single unitig of 3,310,238 bp and the rest three strains (NCTC8678, NCTC8503, and NCTC8797) were assembled into 5–9 unitigs with an N50 of over 2,969,557 bp. These assemblies were run on a High-Performance Computing Cluster using SLURM, utilizing 50 Gb of memory and running parallel on four computing nodes taking ~634.93 min to finish.

### Pangenome assembly and visualisation

The annotation files (general feature format, GFF) of 56 *C. perfringens* genomes were submitted to Roary pangenome pipeline v3.6.1 to perform pangenomic analysis (Page et al., 2015). Roary was run with default parameters. A gene-absence-presence data matrix was subsequently derived and visualised in Phandango genome visualizer (v0.87) (Hadfield and Harris, 2017). Fripan^2^ was used to visualised MDS clustering of isolates (Supplementary Figure 3) and gene-presence-absence tree (Supplementary Figure 4). Recommended R scripts from Roary package were used to generate pangenome stats using R studio(R Development Core Team, 2010; Rstudio Team, 2015).

### Core-genome alignment and construction of phylogenetic trees

Core-genome alignment generated using MAFFT v7.305b (Katoh and Standley, 2013) was used as input for Seaview v4.0 (Gouy et al., 2010) to construct core-genome phylogeny. Neighbour-Joining tree (NJ) was built using Jukes-Cantor model of DNA evolution on a set of 1,000 bootstrap replicates; Maximum-Likelihood (ML) tree was constructed using PhyML v3.0 (Guindon et al., 2010), GTR model and supported by Approximate Likelihood Ratio Test (aLRT). Interactive Tree of Life (iTOL) was used as a tree editor to assign clade and annotate metadata (Letunic and Bork, 2016).

### Alignment-free whole-genome phylogeny

CVTree standalone v5.0 was used for constructing alignment-free whole-genome phylogeny with parameters *k* = 6 on amino acid sequences as recommended for prokaryotes and ATCC13124 was selected as the reference sequence (Qi et al., 2004). All genomes were subject to Prokka annotation prior to building phylogeny to obtain annotated FASTA files (genes in amino acid sequences) (Seemann, 2014). Webtool iTOL was used for phylogeny annotation and editing (Letunic and Bork, 2016).

### 16S rRNA gene alignment, phylogeny, and pair-wise blast

This study employs BAsic Rapid Ribosomal RNA Predictor (Barrnap) v0.5 as 16S rRNA gene predictor (Seemann, 2013). In-house Perl scripts were utilised for extracting the maximum-length 16S rRNA sequence on the positive strands predicted by Barrnap on the WGS data in each isolate for optimal consistency and comparison (Supplementary Table 1). Sequence were aligned using MUSCLE v3.8.31 (Edgar, 2004). Phylogenetic trees were generated as described in previous section. *Clostridium* species 16S rRNA gene sequences were retrieved from NCBI databases (Supplementary Table 3). Pair-wise BLASTn was performed using BLAST+ v2.2.30 and in-house Perl scripts (Camacho et al., 2009).

### Functional annotation of genomes (COG)

Core and accessory genes were extracted using in-house Perl scripts based on pangenome data. The Clusters of Orthologous Groups (COG) classification was done on both the extracted datasets using eggnog-mapper (v0.99.3) based on COG and EggNOG databases, and HMMER was used for sequence homology search and COG class assignment on default parameters (Huerta-Cepas et al., 2016, 2017). COG classes assigned to each gene were then extracted using Shell script^3^ and were plotted using GraphPad Prism v5.04. In-house bash scripts and Linux commands were used to explore annotated data on different functional classes.

### *In Silico* analysis of virulence potentials

**Exotoxin profiles**: 23 presently identified toxins and virulent enzymes sequence data from NCBI database were built into database for BLAST query (Supplementary Table 4). BLAST+ v2.2.30 was employed for sequence similarity search (BLASTn) using in-house Perl and bash scripts (Camacho et al., 2009). Heat maps were generated using R heatmap2 package (R Development Core Team, 2010). Individual *C. perfringens* plasmids were retrieved from NCBI database for toxin profiling (Supplementary Table 5). **Antimicrobial resistance**: sequence similarity search was performed as described above using Comprehensive Antimicrobial Resistance Database (CARD) (Jia et al., 2017). **Prophage profiles**: sequence similarity search was performed as described above using viral databases retrieved from NCBI (March 2017). Percentage identity of 90% was applied for secondary BLASTn filter. **BLAST parameters**: Double-filter parameters were implemented for screening—expect value of 1e-10 and percentage identity of >80% for sequence homology inference unless otherwise indicated.

### CRISPR array detection

CRISPR were predicted using MinCED v0.1.6, a tool derived from CRT (Bland et al., 2007; Angly and Skennerton, 2011). Default parameters were implemented as follows: minimum number of repeats a CRISPR must contain–3, minimum length of CRISPR repeats–23, maximum CRISPR spacers–26, maximum length of CRISPR spaces–50. Counts were parsed in custom bash scripts and visualised using GraphPad Prism v5.04.

### Multiple circular genome comparison

Circular whole genome comparisons were generated and visualised using BLAST Ring Image Generator (BRIG) v0.95 (Alikhan et al., 2011). Percentage identity cut-offs were indicated in the graph legends.

## Author contributions

LH conceived the study and RK, SC, and LH conceptualised the study. RK and SC designed and performed all bioinformatics analysis, visualised all data, and co-wrote the manuscript. PP and SA contributed to the sequencing and assembly work, and co-wrote the manuscript. LH co-wrote and revised the manuscript. All authors have read and approved the final manuscript.

### Conflict of interest statement

The authors declare that the research was conducted in the absence of any commercial or financial relationships that could be construed as a potential conflict of interest.

## Footnotes

^1^NCTC 3000 Project website: www.sanger.ac.uk/resources/downloads/bacteria/nctc/

^2^Fripan website: drpowell.github.io/FriPan/

^3^Public repository: github.com/raymondkiu/eggnog-mapper_COGextraction
