# Supplementary material for: Probing Genomic Aspects of the Multi-Host Pathogen Clostridium perfringens Reveals Significant Pangenome Diversity, and a Diverse Array of Virulence Factors
Source: Front Microbiol. 2017 Dec 12;8:2485. doi: 10.3389/fmicb.2017.02485 (PMC5733095; doi:10.3389/fmicb.2017.02485)
Supplement: Supplementary file 2 [file Presentation1.PDF]

Supplementary Material

Probing genomic aspects of the multi-host pathogen *Clostridium perfringens* reveals significant pangenome diversity, and a diverse array of virulence factors

Raymond Kiu, Shabhonam Caim, Sarah Alexander, Purnima Pachori, Lindsay J. Hall*

*__Correspondence:__

Lindsay J. Hall: lindsay.hall@quadram.ac.uk

Supplementary Figure 1

Supplementary Figure 2

Supplementary Figure 3

Supplementary Figure 4

Supplementary Figure 5

Supplementary Figure 6

Supplementary Table 1

Supplementary Table 2 (on a separate Excel sheet)

Supplementary Table 3

Supplementary Table 4

Supplementary Table 5.

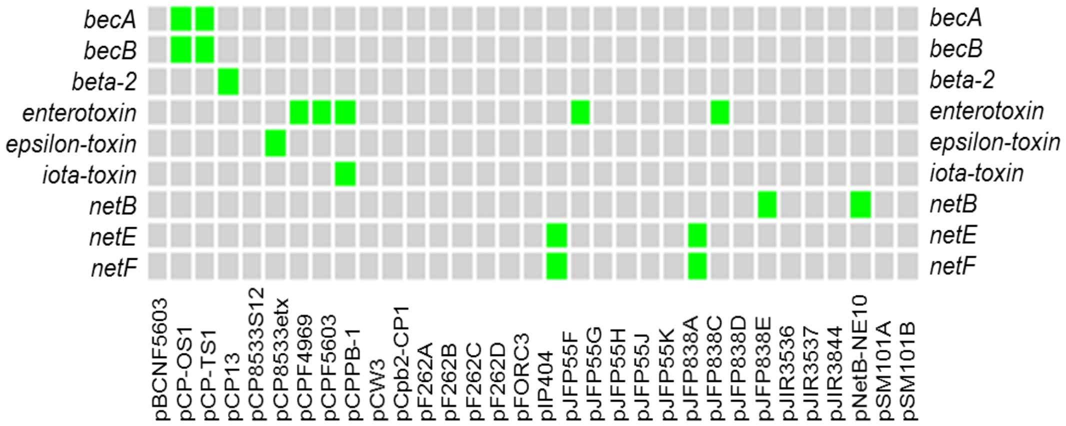

Supplementary Figure 1. Toxins present in the *C. perfringens* plasmids. Gene present: light green; gene absent: grey.

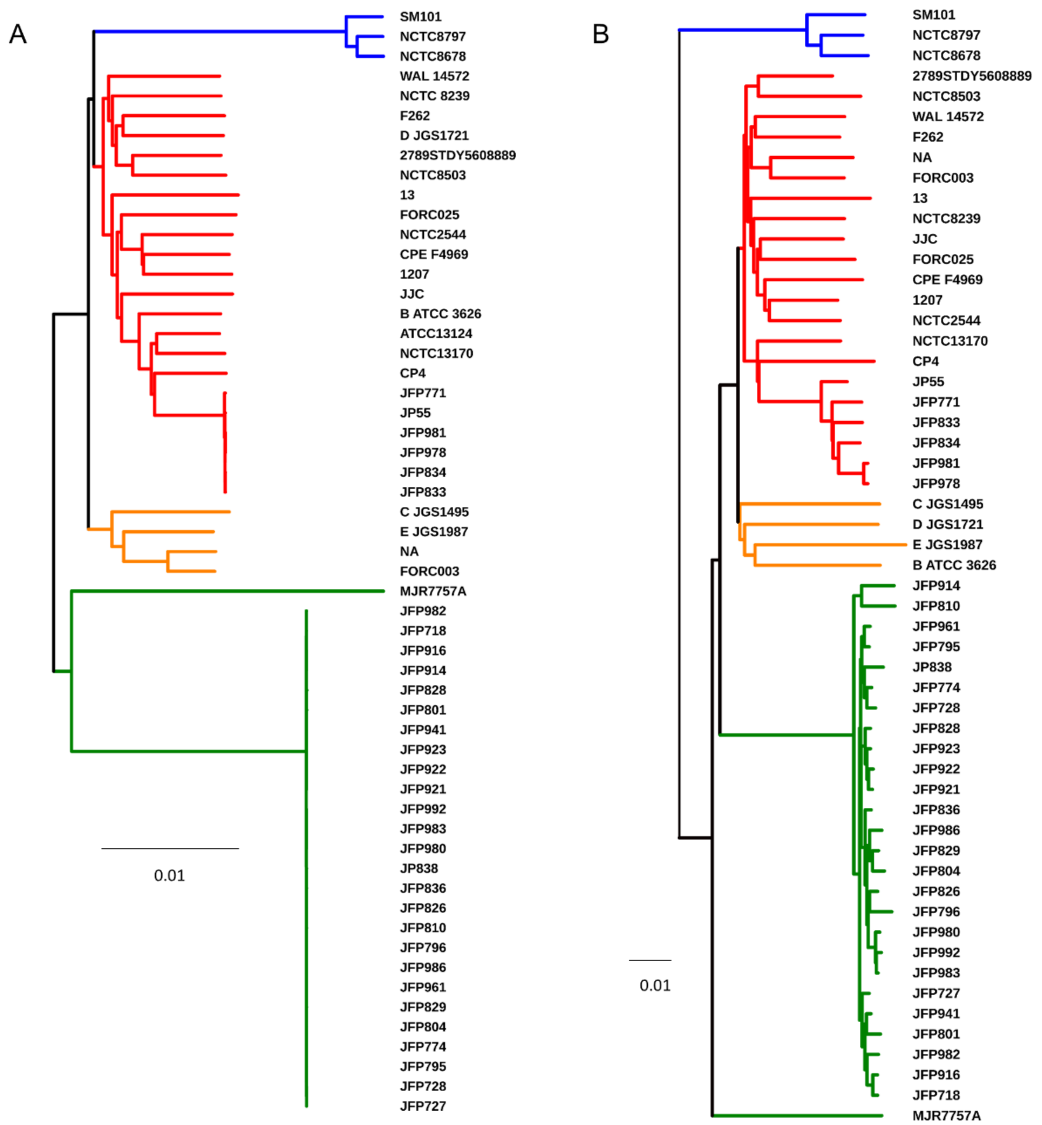

Supplementary Figure 2. (A) Core-genome based phylogeny of 56 *C. perfringens* strains (NJ tree supported by 1000 bootstrap replicates). (B) Whole-genome-based alignment-free phylogeny (CVTree) of 56 *C. perfringens* strains. Colour-coded clades correspond to clade assignment in **Figure 3** for clarity of visualization.

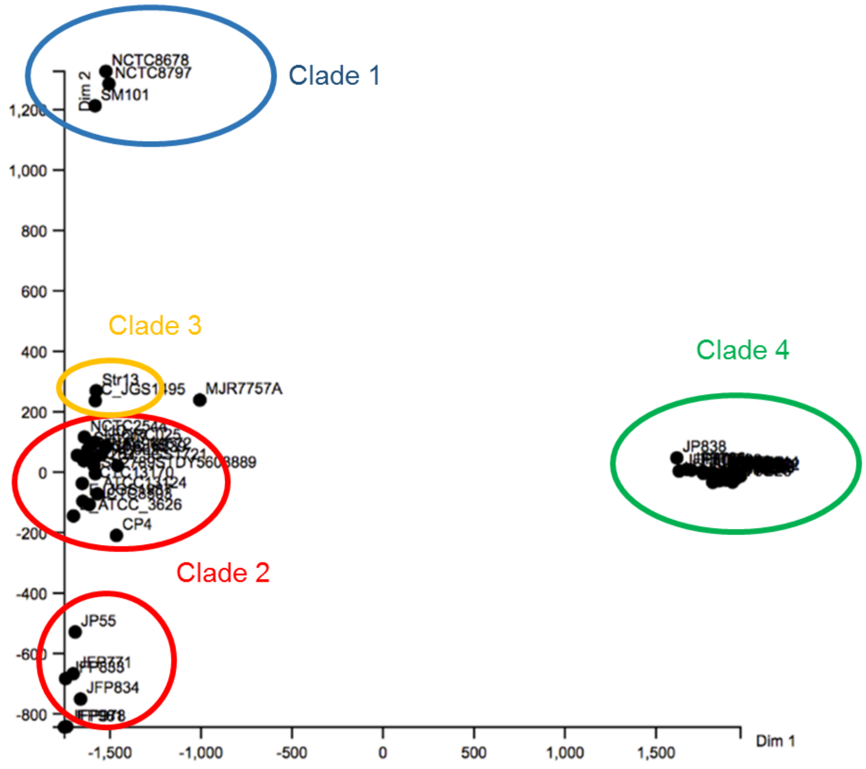

Supplementary Figure 3. Multi-Dimensional Scaling (MDS) plot based on genes-absence-presence profile in the pan genome of 56 *C. perfringens* isolates. Colour-labeling and clade assignments correspond to core-genome phylogeny in **Figure 3**.

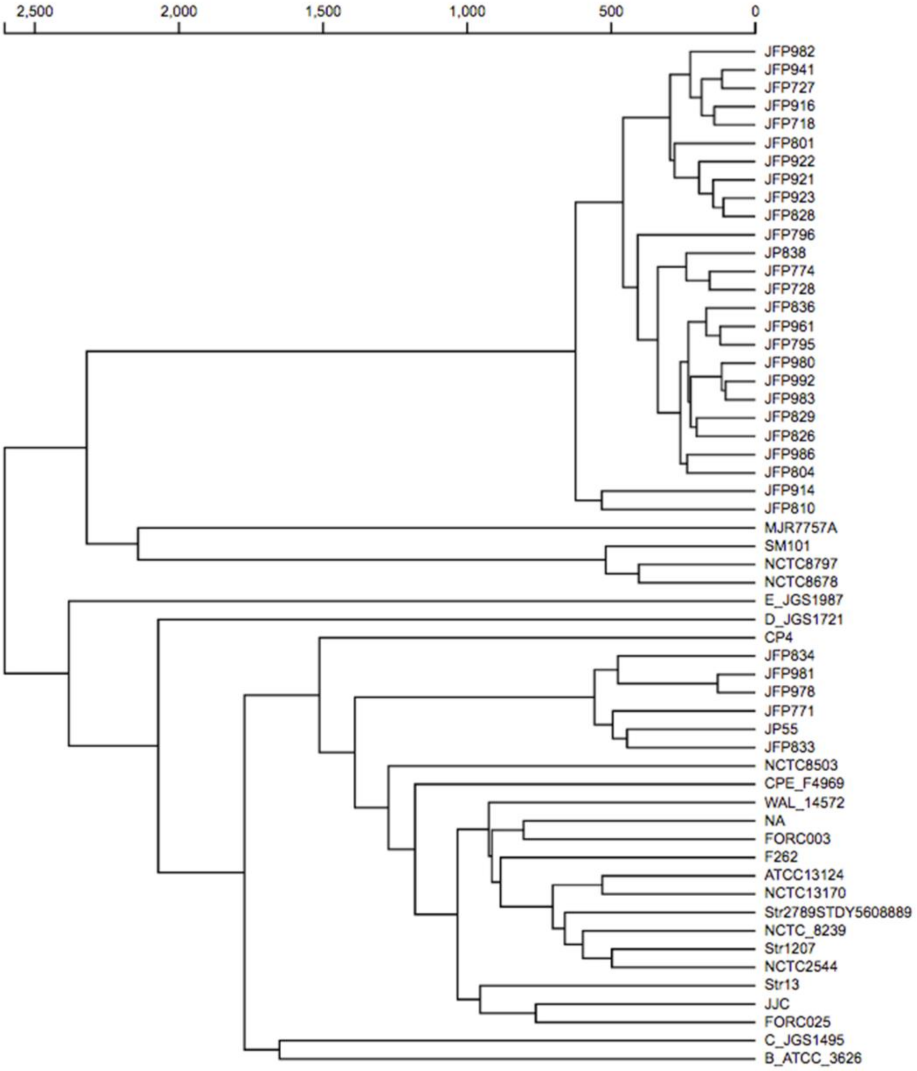

Supplementary Figure 4. Gene-absence-presence based phylogenetic tree of *C. perfringens*.

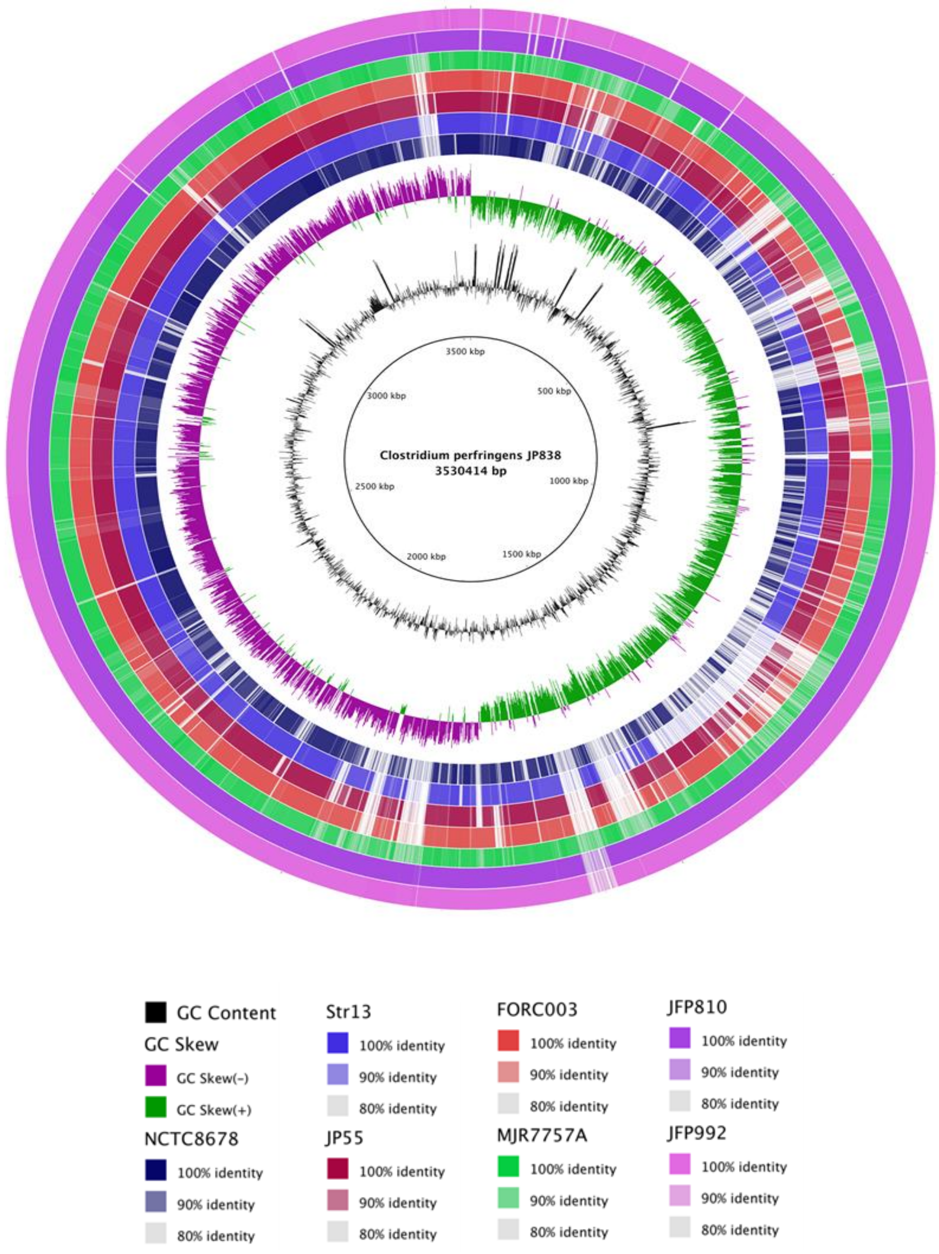

Supplementary Figure 5. Circular whole genome similarity comparison of 8 *C. perfringens* strains representing different clades in the core-genome phylogeny (from inner circle): NCTC8678 (dark blue): Clade 1; Str13 (violet): Clade 2; JP55 (red): Clade 2; FORC003 (orange): Clade 3; MJR7757A (green): Clade 4; JFP810 (purple): Clade 4; JFP992 (pink): Clade 4. Diagram represents BLASTn results of each genome against *C. perfringens* JP838 as reference genome. Relative shading density of coloured blocks (dark to light) indicates relative levels of nucleotide similarity, faded blocks represent low identity regions (<80%).

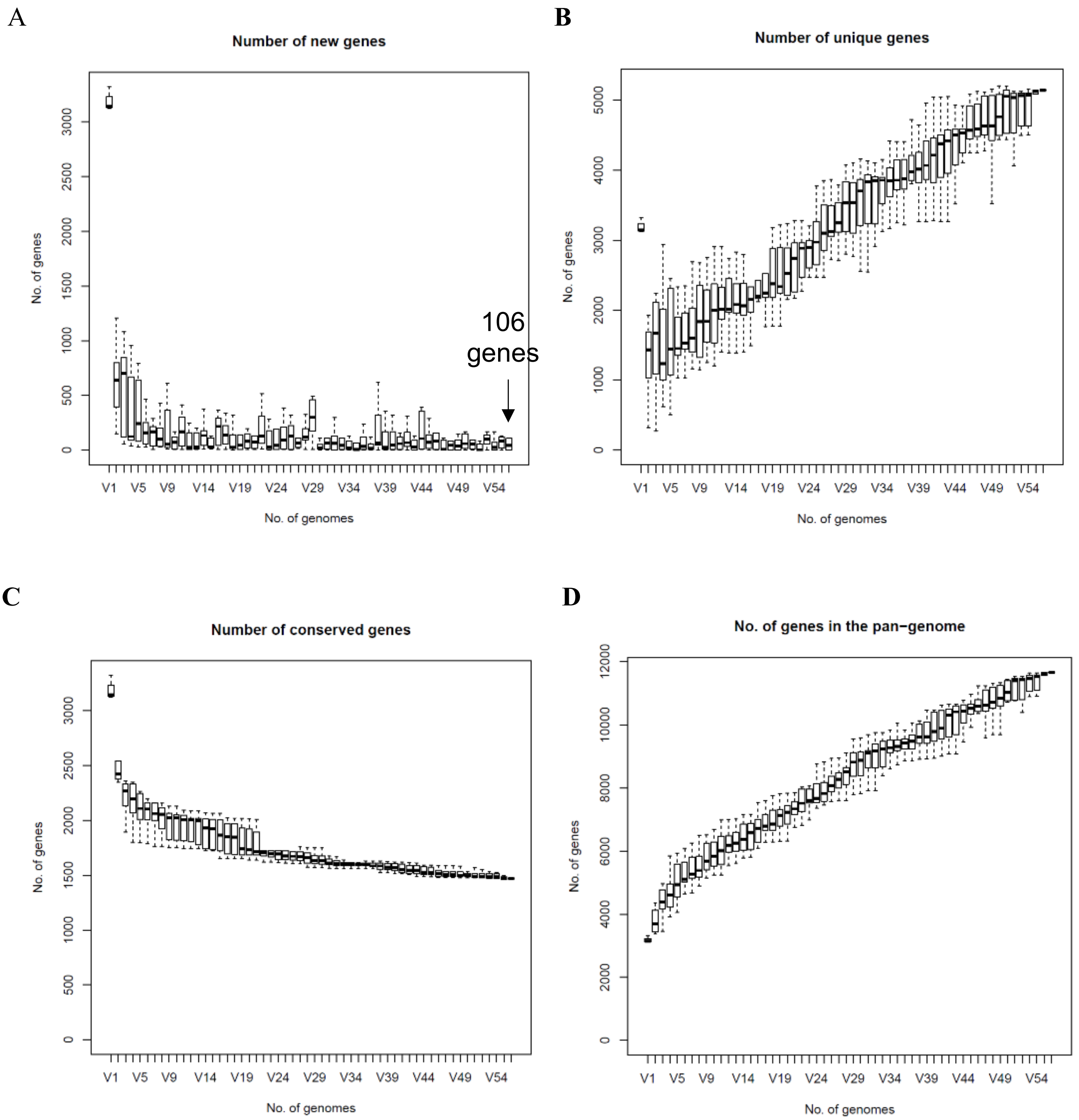

Supplementary Figure 6. Pan-genome computational statistics. (A) Box plot of new genes added to the pan-genome iteration per additional strain. New genes do not converge to zero upon addition of genome at *n*=56, an average of ***106 genes*** are contributed to the gene repertoire. (B) Calculation of unique gene addition. (C) Calculation of conserved genes per genome addition to the gene pool. (D) Number of genes in the pan-genome over 56 genome additions.

Supplementary Table 1. Predicted 16S rRNA gene length (bp) of 38 selected *Clostridium perfringens* isolates using Barrnap.

	Isolates	Predicted 16S rRNA gene length (bp)
1	Str1207	1242
2	Str13	1513
3	NA	1511
4	ATCC13124	1511
5	B_ATCC3626	960
6	C_JGS1495	1511
7	CPE_F4969	1318
8	D_JGS1721	810
9	E_JGS1987	1511
10	F262	1511
11	FORC003	1511
12	FORC025	1511
13	JFP718	1038
14	JFP774	1165
15	JFP796	1511
16	JFP804	1445
17	JFP810	856
18	JFP826	902
19	JFP833	1511
20	JFP836	1511
21	JFP914	1511
22	JFP916	1469
23	JFP922	865
24	JFP923	1511
25	JFP978	1511
26	JFP980	1511
27	JFP981	1104
28	JFP983	1108
29	JFP992	932
30	JP55	1511
31	JP838	1511
32	NCTC8239	878
33	SM101	1511
34	NCTC13170	1511
35	NCTC2544	1511
36	NCTC8503	1511
37	NCTC8678	1511
38	NCTC8797	1511
	**Summary: 38 isolates**	**Min: 810bp**<br>**Max: 1513bp**<br>**Average: 1338bp**

Supplementary Table 3. Accessions of *Clostridium* isolates 16S rRNA gene sequence data used in this study.

	Isolate	Accession
1	*Clostridium rectum* NCIMB10651	NR_119089.1
2	*Clostridium carnis* ATCC25777	NR_044716.2
3	*Clostridium scindens* JCM10421 I-10	AB020730.1
4	*Clostridium difficile* JCM5248	AB632379.1
5	*Clostridium bifermentans* JCM1386	AB618787.1
6	*Clostridium sordellii* ATCC9714	AB075771.1
7	*Clostridium novyi* NT	NC_008593.1
8	*Clostridium tetani* HT1	DQ978212.1
9	*Clostridium pasteurianum* JCM1408	AB536773.1
10	*Clostridium sporogenes* ZE1	LN870314.1
11	*Clostridium botulinum* ATCC3502	NC_009495.1
12	*Clostridium paraputrificum* DSM2630	X73445.1
13	*Clostridium butyricum* JCM1391	AB595129.1
14	*Clostridium botulinum* E133	JN617091.2
15	*Clostridium cadaveris* JCM1392	NR_104695.1
16	*Clostridium sartagoforme* KAR69	KR055029.1
17	*Clostrdiium isatidis* MV1	LN881568.1
18	*Clostridium tertium* ATCC14573	AJ245413.1
19	*Clostridium chauvoei* 2585	NR_026013.1
20	*Clostridium septicum* JCM8147	LC019773.1
21	*Clostridium sardiniense* DSM2632	NR_041006.1
22	*Clostridium fallax* JCM1398	LC036315.1
23	*Clostridium cellulovorans* 743B	NR_102875.1
24	*Clostridium celatum* JCM1394	AB971795.1
25	*Clostridium baratii* JCM1385	LC036313.1
26	*Clostridium absonum* DSM599	X77842.1

Supplementary Table 4. Toxinome custom database and relevant details including virulence and accessions used in this study.

	Genes	Protein encoded	Alternative name	Virulence	NCBI Accession	References
**1**	*plc/cpa*	Phospholipase	Alpha-toxin	Hydrolyse cell membrane	D63911.1	Titball et al. (1999)
**2**	*cpe*	Enterotoxin	-	Disintegration of tight-junctions	M98037.1	Miyamoto et al. (2011)
**3**	*ccp*	Clostripain	-		NC_003366.1	Shimizu et al. (2002)
**4**	*colA*	Microbial collagenase	Kappa-toxin	Digestion of collagen	D13791.1	Matsushita et al. (1994)
**5**	*nanI*	Sialidase	-	Sialidase	NC_003366.1	Shimizu et al. (2002)
**6**	*nanJ*	Exo-alpha-sialidase	-	Sialidase	NC_003366.1	Shimizu et al. (2002)
**7**	*nanH*	Neuraminidase	-	Sialidase	Y00963.1	Roggentin et al. (1988)
**8**	*cpb*	Beta-toxin	-	Pore-formation	KP064410.1	Theoret et al. (2015)
**9**	*cpb2*	Beta-2 toxin	-	Pore-formation	L77965.1	Gibert et al. (1997)
**10**	*pfo*	Perfringolysin O	Theta-toxin	Pore-formation	DQ673100.1	Ohno-Iwashita et al. (1986)
**11**	*nagH*	Hyaluronidase	Mu-toxin	Digestion of connective tissue	NC_003366.1	Shimizu et al. (2002)
**12**	*etx*	Epsilon-toxin	-	Pore-formation	M95206.1	Nagahama et al. (2011)
**13**	*iap*	Iota-toxin component Ia	-	Pore-formation	NC_015712.1	Miyamoto et al. (2011)
**14**	*ibp*	Iota-toxin component Ib	-	Pore-formation	NC_015712.1	Miyamoto et al. (2011)
**15**	*netB*	Pore-forming toxin	-	Cytolysis	FJ189503.1	Fernandes da Costa et al. (2014)
**16**	*becA*	Binary enterotoxin component a	-	Enterotoxin	NC_023918.1	Yonogi et al. (2014)
**17**	*becB*	Binary enterotoxin component b	-	Enterotoxin	NC_023918.1	Yonogi et al. (2014)
**18**	*tpeL*	Peptidase	-	Cytotoxic	EU848493.1	Jiang et al. (2009)
**19**	*cpd*	Delta-toxin	-	Haemolysis	EU652406.1	Manich et al. (2008)
**20**	*netE*	NE Toxin netE	-	Cytolysis	KJ606985	Unpublished
**21**	*netF*	NE Toxin netF	-	Cytolysis	KJ606986	Unpublished
**22**	*netG*	NE Toxin netG	-	Cytolysis	KJ606987	Unpublished
**23**	*lam*	Lambda toxin	-	Potent protease	AJ439340	Unpublished

Supplementary Table 5. Details of retrieved plasmids sequences on NCBI databases used in this study.

	Plasmid	Accession
1	pBCNF5603	AB189671.1
2	pCP13	AP003515.1
3	pCP8533etx	AB444205.1
4	pCP8533S12	AB736082.1
5	pCpb2-CP1	JQ655732.1
6	pCPF4969	AB236336.1
7	pCPF5603	AB236337.1
8	pCP-OS1	AP013033.1
9	pCPPB-1	AB604032.1
10	pCP-TS1	AP013034.1
11	pCW3	DQ366035.1
12	pF262A	CM001478.1
13	pF262B	CM001479.1
14	pF262C	CM001480.1
15	pF262D	CM001481.1
16	pFORC3	CP009558.1
17	pIP404	M32882.1|
18	pJFP55F	CP013041.1
19	pJFP55G	CP013042.1
20	pJFP55H	CP013043.1
21	pJFP55J	CP013044.1
22	pJFP55K	CP013045.1
23	pJFP838A	CP013615.1
24	pJFP838C	CP013040.1
25	pJFP838D	CP013039.1
26	pJFP838E	CP013038.1
27	pJIR3536	JN689219.1
28	pJIR3537	JN689220.1
29	pJIR3843	JN689218.1
30	pJIR3844	JN689217.1
31	pNetB-NE10	JQ655731.1
32	pSM101A	CP000313.1
33	pSM101B	P000314.1

References

Fernandes da Costa, S.P., Savva, C.G., Bokori-Brown, M., Naylor, C.E., Moss, D.S., Basak, A.K., et al. (2014). Identification of a key residue for oligomerisation and pore-formation of Clostridium perfringens NetB. *Toxins (Basel)* 6(3)**,** 1049-1061. doi: 10.3390/toxins6031049.

Gibert, M., Jolivet-Reynaud, C., and Popoff, M.R. (1997). Beta2 toxin, a novel toxin produced by Clostridium perfringens. *Gene* 203(1)**,** 65-73.

Jiang, Y.F., Kulkarni, R.R., Parreira, V.R., and Prescott, J.F. (2009). Immunization of Broiler Chickens Against Clostridium perfringens-Induced Necrotic Enteritis Using Purified Recombinant Immunogenic Proteins. *Avian Diseases* 53(3)**,** 409-415.

Manich, M., Knapp, O., Gibert, M., Maier, E., Jolivet-Reynaud, C., Geny, B., et al. (2008). Clostridium perfringens delta toxin is sequence related to beta toxin, NetB, and Staphylococcus pore-forming toxins, but shows functional differences. *PLoS One* 3(11)**,** e3764. doi: 10.1371/journal.pone.0003764.

Matsushita, O., Yoshihara, K., Katayama, S., Minami, J., and Okabe, A. (1994). Purification and characterization of Clostridium perfringens 120-kilodalton collagenase and nucleotide sequence of the corresponding gene. *J Bacteriol* 176(1)**,** 149-156.

Miyamoto, K., Yumine, N., Mimura, K., Nagahama, M., Li, J., McClane, B.A., et al. (2011). Identification of novel Clostridium perfringens type E strains that carry an iota toxin plasmid with a functional enterotoxin gene. *PLoS One* 6(5)**,** e20376. doi: 10.1371/journal.pone.0020376.

Nagahama, M., Itohayashi, Y., Hara, H., Higashihara, M., Fukatani, Y., Takagishi, T., et al. (2011). Cellular vacuolation induced by Clostridium perfringens epsilon-toxin. *FEBS J* 278(18)**,** 3395-3407. doi: 10.1111/j.1742-4658.2011.08263.x.

Ohno-Iwashita, Y., Iwamoto, M., Mitsui, K., Kawasaki, H., and Ando, S. (1986). Cold-labile hemolysin produced by limited proteolysis of theta-toxin from Clostridium perfringens. *Biochemistry* 25(20)**,** 6048-6053.

Roggentin, P., Rothe, B., Lottspeich, F., and Schauer, R. (1988). Cloning and sequencing of a Clostridium perfringens sialidase gene. *FEBS Lett* 238(1)**,** 31-34.

Shimizu, T., Ohtani, K., Hirakawa, H., Ohshima, K., Yamashita, A., Shiba, T., et al. (2002). Complete genome sequence of Clostridium perfringens, an anaerobic flesh-eater. *Proc Natl Acad Sci U S A* 99(2)**,** 996-1001. doi: 10.1073/pnas.022493799.

Theoret, J.R., Uzal, F.A., and McClane, B.A. (2015). Identification and characterization of Clostridium perfringens beta toxin variants with differing trypsin sensitivity and in vitro cytotoxicity activity. *Infect Immun* 83(4)**,** 1477-1486. doi: 10.1128/IAI.02864-14.

Titball, R.W., Naylor, C.E., and Basak, A.K. (1999). The Clostridium perfringens alpha-toxin. *Anaerobe* 5(2)**,** 51-64. doi: 10.1006/anae.1999.0191.

Yonogi, S., Matsuda, S., Kawai, T., Yoda, T., Harada, T., Kumeda, Y., et al. (2014). BEC, a novel enterotoxin of Clostridium perfringens found in human clinical isolates from acute gastroenteritis outbreaks. *Infect Immun* 82(6)**,** 2390-2399. doi: 10.1128/IAI.01759-14.
